# NLRP3–GABA signaling pathway contributes to the pathogenesis of impulsive-like behaviors and cognitive deficits in aged mice

**DOI:** 10.1186/s12974-023-02845-3

**Published:** 2023-07-11

**Authors:** Lu-Ying Wang, Xu-Peng Wang, Jin-Meng Lv, Yu-Dong Shan, Shi-Yan Jia, Zhi-Fang Yu, Hui-Tao Miao, Yue Xin, Dong-Xue Zhang, Li-Min Zhang

**Affiliations:** 1 Department of Anesthesia and Trauma Research, Department of Anesthesiology, Hebei Province Cangzhou Hospital of Integrated Traditional and Western Medicine, Cangzhou, China; 2 Department of Anesthesiology, Third Hospital of Hebei Medical University, Shijiazhuang, Hebei China; 3 Department of Anesthesiology, Hebei Province Cangzhou Hospital of Integrated Traditional and Western Medicine, Cangzhou, China; 4 Department of Gerontology, Cangzhou Central Hospital, Cangzhou, China

**Keywords:** NLRP3, Selegiline, Astrocyte, GABA, Perioperative neurocognitive disorders

## Abstract

**Background:**

Perioperative neurocognitive disorders (PND), such as delirium and cognitive impairment, are commonly encountered complications in aged patients. The inhibitory neurotransmitter γ-aminobutyric acid (GABA) is aberrantly synthesized from reactive astrocytes following inflammatory stimulation and is implicated in the pathophysiology of neurodegenerative diseases. Additionally, the activation of NOD-like receptor protein 3 (NLRP3) inflammasome is involved in PND. Herein, we aimed to investigate whether the NLRP3–GABA signaling pathway contributes to the pathogenesis of aging mice’s PND.

**Methods:**

24-month-old C57BL/6 and astrocyte-specific NLRP3 knockout male mice were used to establish a PND model via tibial fracture surgery. The monoamine oxidase-B (MAOB) inhibitor selegiline (1 mg/kg) was intraperitoneally administered once a day for 7 days after the surgery. PND, including impulsive-like behaviors and cognitive impairment, was evaluated by open field test, elevated plus maze, and fear conditioning. Thereafter, pathological changes of neurodegeneration were explored by western blot and immunofluorescence assays.

**Results:**

Selegiline administration significantly ameliorated TF-induced impulsive-like behaviors and reduced excessive GABA production in reactive hippocampal astrocytes. Moreover, astrocyte-specific NLRP3 knockout mice reversed TF-induced impulsive-like and cognitive impairment behaviors, decreased GABA levels in reactive astrocytes, ameliorated NLRP3-associated inflammatory responses during the early stage, and restored neuronal degeneration in the hippocampus.

**Conclusions:**

Our findings suggest that anesthesia and surgical procedures trigger neuroinflammation and cognitive deficits, which may be due to NLRP3–GABA activation in the hippocampus of aged mice.

**Supplementary Information:**

The online version contains supplementary material available at 10.1186/s12974-023-02845-3.

## Introduction

Perioperative neurocognitive disorder (PND), including delirium and cognitive dysfunction, is a serious perioperative complication that affects 19–52% of elderly patients. Age and type of surgery have emerged as risk factors for this disorder [1, 2]. As is well documented, geriatric anxiety and depressive disorders, as well as common neuropsychiatric symptoms of cognitive impairment, severely disrupt the healing process and quality of life in aged patients [3]. Numerous publications have described that the neuroinflammatory response-induced cholinergic imbalance, which purportedly participates in the dementia symptoms of Alzheimer’s disease (AD), may be associated with PND [4, 5]. Furthermore, a study published in the *Lancet* has shown that anesthesia alone has a minimal effect on cognitive function [6]. However, the exact underlying mechanism remains unclear to date.

In the brains of AD patients, glial cells are significantly altered in terms of morphology and gene expression [7]. Astrocytes are reactive, especially in round amyloid plaques, which can be demonstrated by reinforcement strategies and improved glial fibrillary acidic protein (GFAP) expression [8]. Among these glial transmitters, γ-aminobutyric acid (GABA) is the most important inhibitory transmitter in the mammalian brain, and AD patients exhibit a wide range of GABA in the cerebrospinal fluid, although the mechanism by which GABA levels are elevated is not known [9, 10]. It was previously confirmed that a low level of catatonic GABA launch is determined in the hippocampus beneath everyday prerequisites [11]. Interestingly, selective inhibition of astrocytic GABA synthesis or initiation has been reported to be an excellent therapeutic approach for the treatment of AD [12]. Therefore, we assumed that an extraordinary increase in catatonic GABA launch from reactive astrocytes in the hippocampus may also be simultaneously accountable for the technique of PND after surgical exposure.

Studies have established that the progression of numerous neurodegenerative diseases, including PND, is brought about by neuroinflammation [13]. There is a growing body of evidence that indicates that neuroinflammation may arise in the course of neurodegenerative diseases owing to the fact that pro-inflammatory signaling molecules have been recognized in each sufferer and animal model of PND [14, 15]. The nod-like receptor protein 3 (NLRP3) inflammatory vesicles are intracellular protein complexes that play a fundamental role in innate immune perception [16]. In addition, activation of NLRP3 inflammatory vesicles causes caspase-1 maturation, and the conversion of pro-IL-18 to mature and activate IL-18 is dependent on caspase-1 mediation. Recently, we reported that the inhibition of the NLRP3–IL-18 signaling pathway in astrocytes is involved in mood changes following traumatic stress [17]. Besides, the inhibition of NLRP3 inflammasome in the hippocampus alleviated PND in aged mice [18]. Notably, the deficiency in the GABAergic system attenuated macrophage-mediated NLRP3 inflammasome activation [19]. However, whether NLRP3–GABA signaling pathway contributes to the pathogenesis of PND in aged mice remains unknown.

To investigate this hypothesis, we examined an extensively used mannequin of the tibial fracture surgery model of PND in aged mice and sought to investigate whether or not manipulating the NLRP3–GABA signaling pathway is a viable therapeutic approach to relieve PND in aged mice.

## Methods

All experiments involving animals were carried out in accordance with the National Institute of Health Guidelines for the Care and Use of Laboratory Animals. The protocols involving animals were additionally approved by the Animal Review Board of Hebei Province Cangzhou Hospital of Integrated Traditional and Western Medicine.

### Group assignment

**Fig. 1:**
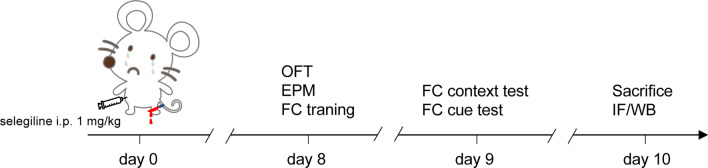
Experimental schematic diagram. Perioperative neurocognitive disorders (PND) were established by tibial fracture surgery (TF) in aged mice. At 8 days after TF exposure, open field test (OFT), elevated plus maze (EPM), and the first stage of fear conditioning (FC) were performed. At 9 days after TF exposure, the second stage of FC was performed. At 10 days after TF exposure, the immunofluorescence (IF) and western blot assay (WB) experiments were performed. Selegiline was administered from postoperative day 0 to 7 once a day. An equal volume of normal saline was used as vehicle

Male C57BL/6 mice and astrocyte-specific NLRP3 knockout mice (24 months old) were used to establish a tibial plateau fracture (TF) model. Prior to surgical treatment, mice were housed in individually ventilated cage systems at a temperature of 23 °C and humidity of 40–60% and allowed water and food ad libitum. In the first stage, wild-type aged mice were randomly assigned to two groups: (1) sham (*n* = 18), (2) TF (*n* = 20); in the second stage, wild-type aged mice were randomly assigned to four groups: (1) sham + vehicle (*n* = 12), (2) TF + vehicle (*n* = 12), (3) sham + selegiline (*n* = 12), (4) TF + selegiline (*n* = 13); in the third stage, wild-type mice and NLRP3-cKO mice were selected and divided into two groups: (1) wild-type (WT) TF (*n* = 15), (2) NLRP3-cKO TF (*n* = 15) (Additional file 1). Selegiline (1 mg/kg, HY-14198, MedChemExpress, USA), a monoamine oxidase-B (MAOB) inhibitor, was administered from postoperative day 1 to 7 once a day. An equal volume of saline was administered as vehicle (Fig. 1).

### TF model

Aged mice were placed in an anesthesia chamber loaded with 7–8% sevoflurane as the induction agent, and after anesthesia, the mice were placed on a heating pad at 36–38 °C for the intervention, and the whole operation was performed under 3–4% concentration of sevoflurane anesthesia. After shaving and disinfection, a 1-cm longitudinal incision was made on the tibial plateau, and then the tibial plateau was fully exposed. During the procedure, the distal patellar ligament stop was located medially, and an 8 mm long, 0.3 mm inner diameter steel needle was inserted perpendicular to the tibial plateau and along the longitudinal axis of the tibial medullary cavity into the tibial medullary cavity. Subsequently, the tibia was cut in 1/2 with surgical scissors. Afterward, lidocaine was added to the incision site following the necessary debridement to stop hemorrhage, and then the wound was closed. We used 0.5% lidocaine 100 µL for local infiltration in all mice that underwent surgery, both in the model and sham groups, to achieve better postoperative analgesia and facilitate recovery. The mice were kept warm after surgery and allowed to wake up naturally. The surgery lasted for approximately 15 min. Mice in the sham group were only anesthetized, shaved, and given a 1-cm tibial plateau incision followed by suturing. After completion of the intervention, mice were inserted into an incubator maintained at 35 °C for 30 min and then back into the cage.

### Open field test (OFT)

At 8 days post-surgery, OFT was used to evaluate the postoperative mobility of the aged mice. The experimental apparatus was a large open-air box (60 cm × 60 cm × 40 cm), and the box was placed in the lower right corner for 5 min with the mouse in the lower right corner. The distance walked and the average speed was recorded during the test and analyzed by the Super Maze Tracking System (XR-XZ301, Shanghai Xinruan Software Co., Ltd., Shanghai, China). After the experiment, the apparatus was cleaned and sterilized, and the experimental chamber was wiped with 75% alcohol to eliminate the previous olfactory cues of the mouse. During the evaluation of all behavioral tests, videos were evaluated by scientists who did not observe the behavioral tests and were blinded to the group assignment of animals.

### Elevated plus maze (EPM)

One hour following OFT, an elevated cross-maze experiment, which relies on the preference of mice for a dark and closed environment, was used to observe impulsive-like behaviors in mice. A device with two open arms and two closed arms raised 75.5 cm above the ground was used. There were 51 cm of arms, 11.5 cm of width, and 40.5 cm of walls. Each mouse was given five minutes to explore the maze. Using the Super Maze tracking system, the ratio between the time spent in the open arm and the central area to the total time of the experiment was analyzed, and the trajectories of the mice were recorded.

### Fear conditioning (FC)

Rodents exhibit characteristic immobility in response to fear [19]. The FC test was used to assess memory deficits in mice. One hour after the EPM experiment, the FC training phase was conducted. The mice were placed in a conditioned fear test chamber (ZHONGSHI SCIENCE & TECHNOLOGY, Beijing, China) and received 180 s of environmental stimulation, followed by 30 s of acoustic stimulation (70 dB, 3 kHz), followed by 2 s of electric shock (0.75 mA), and the above training was performed in triplicate. In the test chamber, the mice were removed, and the chamber was thoroughly cleaned with 75% alcohol for the next animal to be trained. Tests were administered at the same time after training and included a background association test and a sound association test. The mice were initially placed in the same test chamber as during training for 180 s. Background association stimulation was performed, and the conditioned fear analysis software automatically recorded the immobility of the mice and the time spent maintaining immobility. Meanwhile, the sound association test was performed 2 h after the background association stimulation. The test chamber environment was first modified by replacing the gridded floor inside the test chamber with a smooth plastic sheet and subsequently adding a colored plastic sheet on the diagonal of the test chamber, which converted the rectangular test chamber into a triangular one. The mice were placed in the modified chamber and subjected to 180 s of environmental stimulation, followed by 180 s of acoustic stimulation (70 dB, 3 kHz), and immobility and duration of immobility were recorded. The mice’s memory abilities were examined by analyzing the time of immobility after environmental stimulation and sound stimulation.

### Immunofluorescence

Under sevoflurane anesthesia, mice were perfused with ice-cold saline and 10% neutral buffered formalin via the left ventricular aorta. Mice were decapitated and their brain tissue carefully removed. A mouse brain slicer was then used to make vertical slices along 2 mm posterior to the fontanelle of the mouse brain to a thickness of approximately 1 mm, followed by dehydration and embedding. The tissue was collected, and the tissue sections were sliced into 4-μm-thick cubes, which were embedded in paraffin wax. Antigen repair was performed after treatment with sodium citrate and then blocked with a Quickblock (Cat#P0260, Beyotime, Shanghai, China). The slices were incubated with monoclonal mouse against GFAP (1:100, Cat#AF0156; Beyotime), NLRP3 (1:100, Cat#K008087P; Solarbio, Beijing, China), GAD65 (1:100, Cat#ab239372; Abcam, Cambridge, UK), VGLUT2 (1:100, Cat#ab216463; Abcam), NeuN (1:500, Cat#ab104224; Abcam), GABA (1:100, bs-2252R, bioss, MA, USA), Glutamate (1:100, Cat#G6642; Sigma, MO, USA), IL-18 (1:100, Cat#AF7266; Beyotime), IL-1β (1:100, Cat#K101295P; Solarbio) and cleaved-caspase1 (1:100, Cat#AF4022; Affinity, USA) overnight at 4 °C. On the second day, the slices were incubated with secondary antibodies (CyTM3-conjugated donkey anti-goat IgG, 1.5 mg/mL, Cat#A0502, Beyotime; CyTM3-conjugated goat anti-mouse IgG, 1.5 mg/mL, Cat#A0521, Beyotime; and FITC-conjugated goat anti-rabbit IgG, 1.5 mg/mL, Cat#A0562, Beyotime) in the dark for 1 h. Nuclei were stained with 5 μg/mL of DAPI (Cat#P0131; Beyotime) after rinsing with PBS. Using a fluorescence microscope (RVL-100-M; Echo, USA), a pathologist blinded to grouping assessed the above slices. An average of six fields with a magnification of 200 were randomly selected on six slices. The intensity of NeuN, GFAP, GAD2, and VGLUT2, as well as GABA-, glutamate-, cleaved caspase-1-, IL-1β-, and IL-18-occupied areas in GFAP-positive cells were analyzed by Image-Pro Plus 6.0 (NIH, Bethesda, MD, USA). The co-stained area of positive cells was calculated using Image-Pro Plus 6.0 (NIH, Bethesda, MD, USA). Select the color threshold (Hue from 30 to 80, Brightness from 81 to 255) and click Select. The co-stained parts are marked in yellow. Then set the scale, change the unit from pixel to um, and finally click Analyze–Measure to get the fluorescence co-stained area.

### Western blot assay

The left ventricular aortae of mice were perfused with 0.9% cold saline under 7–8% sevoflurane anesthesia. Afterward, the mouse brains were dissected, the hippocampus was isolated, and total protein was extracted. Samples (30 µg protein) were added to 12% SDS-PAGE gels for separation. The separated proteins were transferred to PVDF membranes. The membranes were then blocked by a Quickblock (Cat#P0252, Beyotime) at room temperature for 10 min. Subsequently, the PVDF membranes were incubated overnight at 4 °C with primary antibodies including IL-18 (1:1000, Cat#AF7266; Beyotime), IL-1β (1:1000, Cat#K101295P; Solarbio), MAP2 (1:1000, Cat#AP63321; Abcepta; CA, USA) and PSD95 (1:1000, WL05046; Wanleibio; Shenyang; China). On the second day, after incubation with goat anti-rabbit secondary antibodies (1:1000, Cat#A0208, Beyotime) overnight at 25 °C for 1 h, the membranes were incubated with ECL (Cat#P0018AM, Beyotime) for 5 min. α-Tubulin (1:1000, Cat#AF0001; Beyotime) was used as an internal reference. OD values were calculated using ImageJ software (National Institutes of Health, Bethesda, MD, USA). All experiments were performed in triplicates (Additional file 1).

### Statistical analysis

Based on the available data, the incidence of PND after major arthroplasty was 16–45%, and we predetermined the sample size for total efficacy *α* = 0.05 and *β* = 0.8 [20]. Data were expressed as mean ± standard deviation (SD). All statistical analyses were performed using GraphPad Prism 8 software (GraphPad Software Inc., San Diego, CA, USA). To evaluate differences between data following a normal distribution, a one-way analysis of variance (ANOVA) was conducted, followed by Tukey’s multiple comparison test. For non-normal distributions, the Kruskal–Wallis test was conducted, followed by a Dunn–Bonferroni test. *P* < 0.05 was considered statistically significant.

## Results

### Aged mice exhibit impulsive-like behaviors and cognitive deficits post-surgery in aged mice

This study comprised 117 mice. Two mice in the TF group died from postoperative osteomyelitis, and one mouse in the TF + selegiline group died from an intraperitoneal drug injection error.

**Fig. 2:**
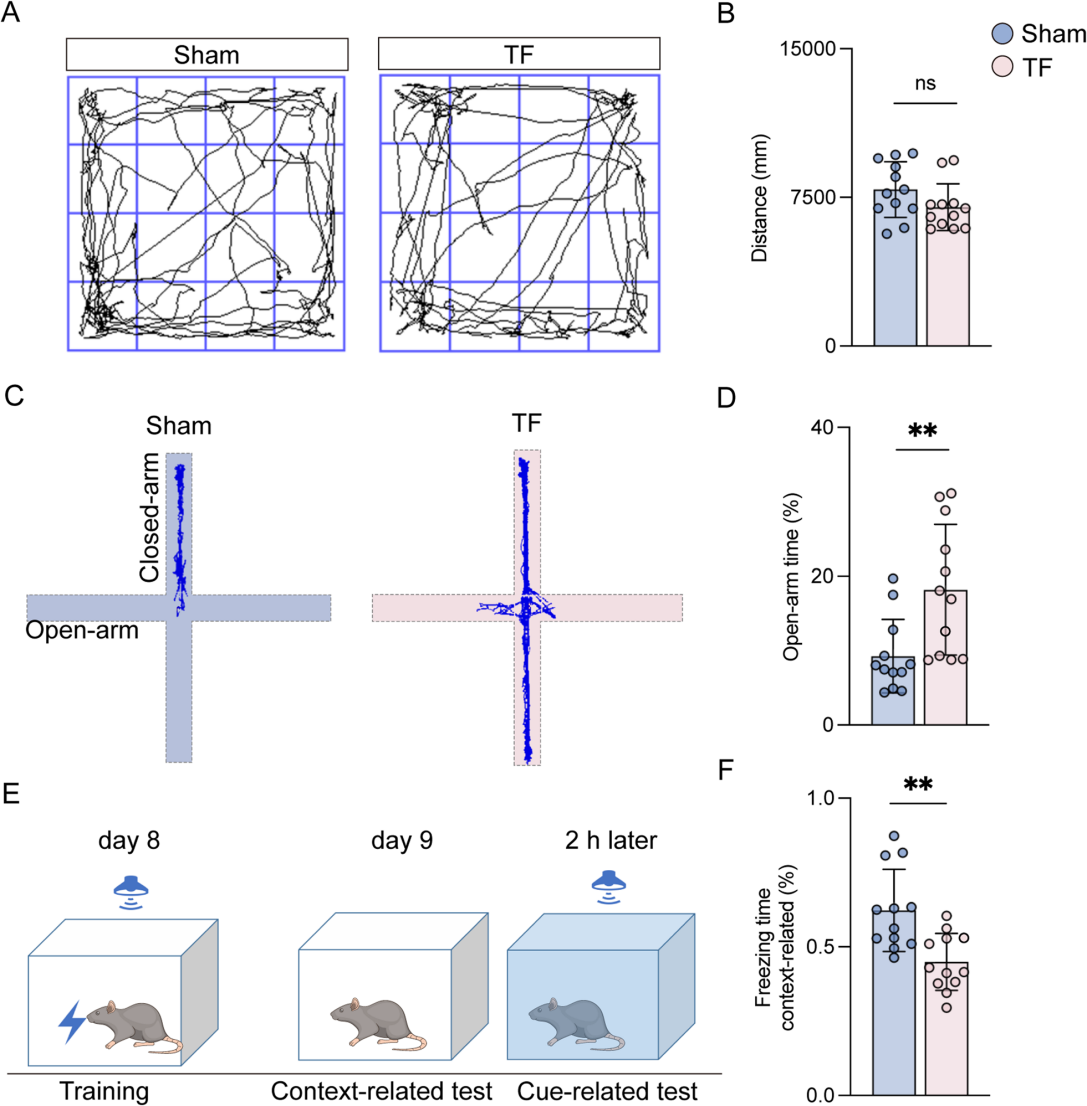
Anesthesia and surgery caused impulsive-like behaviors and cognitive deficits in aged mice. **A** Computer printouts showing the shifting trajectories of each group in the OFT at 8 days after surgical exposure. **B** The total distance for each group at 8 days after surgical exposure. **C** Computer printouts showing the shifting trajectories of each group in the EPM at 8 days after surgical exposure. **D** The time spent in the open arm for each group at 8 days after surgical exposure. **E** FC test experimental schematic diagram. **F** Freezing time during the FC test results caused by the indicated stimuli. Data are presented as the mean ± SD (*n* = 12 mice/group). Data were analyzed by one-way ANOVA with Tukey’s multiple comparison test or Kruskal–Wallis and Dunn’s multiple comparison test. **P < 0.01

The result of OFT revealed that there were no significant differences in total distance and average speed between aged mice exposed to sham and TF, which indicated that TF surgery did not significantly alter locomotor ability (Fig. 2A, B). Interestingly, in the EPM, there was significant increase in time spent in the open arm of mice in the TF group, and they exhibited decreased context-related freezing time in the FC compared with the sham group (Fig. 2C–F). These results suggested that anesthesia and surgery resulted in impulsive-like behaviors and cognitive deficits in aged mice.

**Fig. 3:**
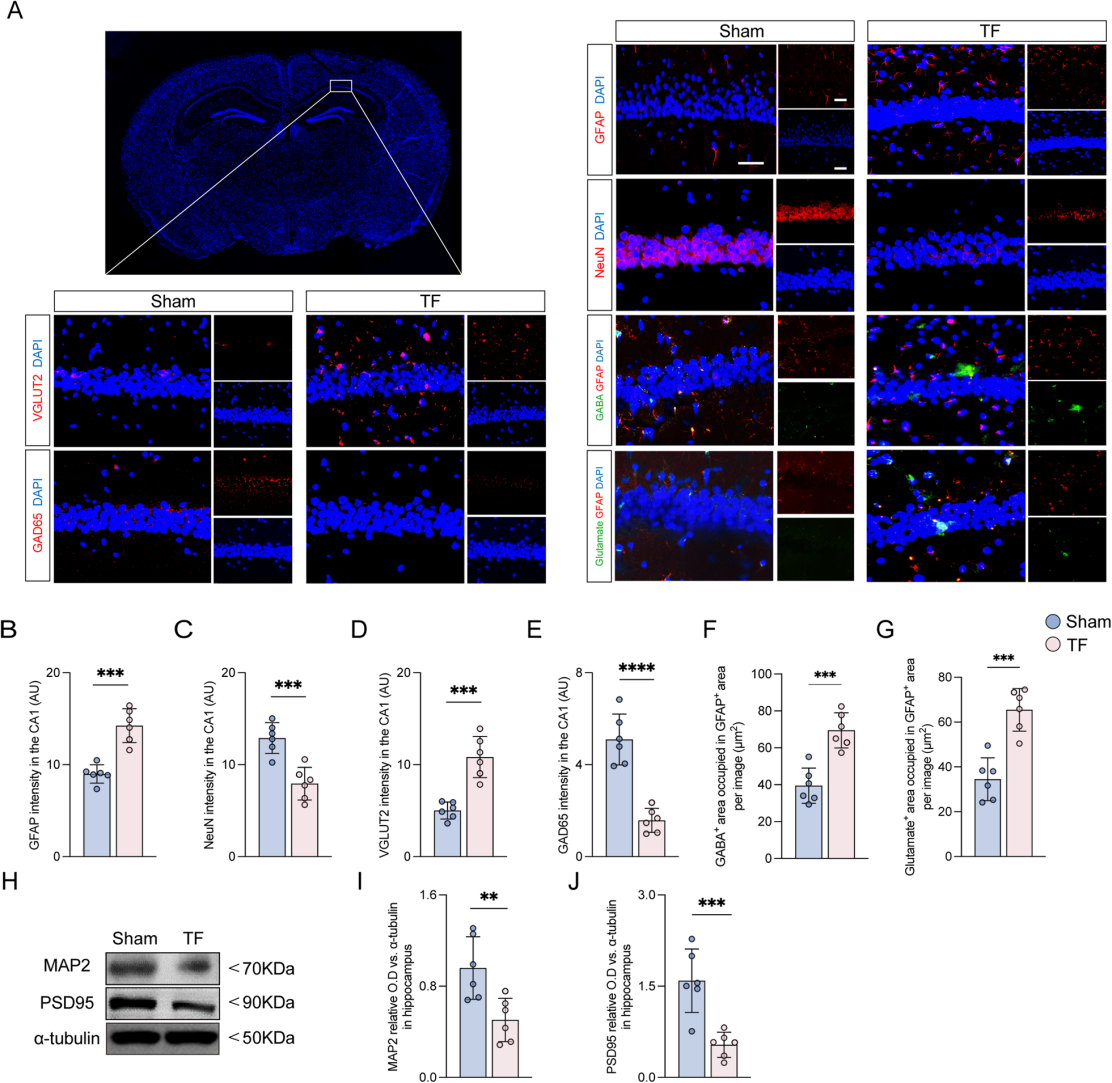
Anesthesia and surgery lead to neuronal damage, reactive astrocytes, and increased excitatory neurons. **A** Representative photomicrographs of GFAP-, NeuN-, VGLUT2-, GAD65-, GABA- and glutamate-positive cells in the CA1 of the hippocampus. Scale bar = 50 μm. **B**–**E** The intensity of GFAP, NeuN, VGLUT2 and GAD65 was quantified in each group. **F**, **G** Co-stained area of GABA- and GFAP-positive cells, and glutamate- and GFAP-positive cells in the CA1 of the hippocampus. **H** Representative western blot of MAP2 and PSD95. **I**, **J** The optical density values of MAP2 and PSD95 in the hippocampus. Data are presented as the mean ± SD (*n* = 6 mice/group). Data were analyzed by one-way ANOVA with Tukey’s multiple comparison test or Kruskal–Wallis and Dunn’s multiple comparison test. ****P < 0.0001; ***P < 0.001; **P < 0.01; *P < 0.05

Given the prominent role of the hippocampus in PND [21], we focused on pathological changes in this region 10 days after surgical exposure. The results demonstrated that NeuN intensity was significantly decreased, but GFAP intensity was dramatically increased in mice exposed to TF surgery compared to sham (Fig. 3A–C). Intriguingly, GABAergic neurons, as indicated by GAD65 intensity, were reduced, but glutamatergic neurons, as indicated by VGlut2 intensity, were elevated in mice exposed to TF surgery compared to sham (Fig. 3A, D, and E). Besides, GABA and glutamate expressions in astrocytes, as determined by GABA- and glutamate-occupied regions in GFAP-positive cells, were upregulated in the CA1 of mice exposed to TF surgery compared to sham surgery (Fig. 3A, F, and G). Previous studies have reported that cognitive deficits were closely associated with decreased dendrites and post-synapse proteins [22, 23]. The results of the western blot showed that the expression levels of MAP2 and PSD95 were all decreased in aged mice exposed to surgical exposure compared with sham at 10 days after surgery (Fig. 3H–J). These results implied that anesthesia and surgery led to neuronal damage, reactive astrocytes, and increased excitatory neurons but decreased inhibitory neurons in the hippocampus, which may be associated with impulsive-like behaviors and cognitive deficits.

### Selegiline administration ameliorates impulsive-like behaviors but not cognitive impairment post-surgery in aged mice

**Fig. 4:**
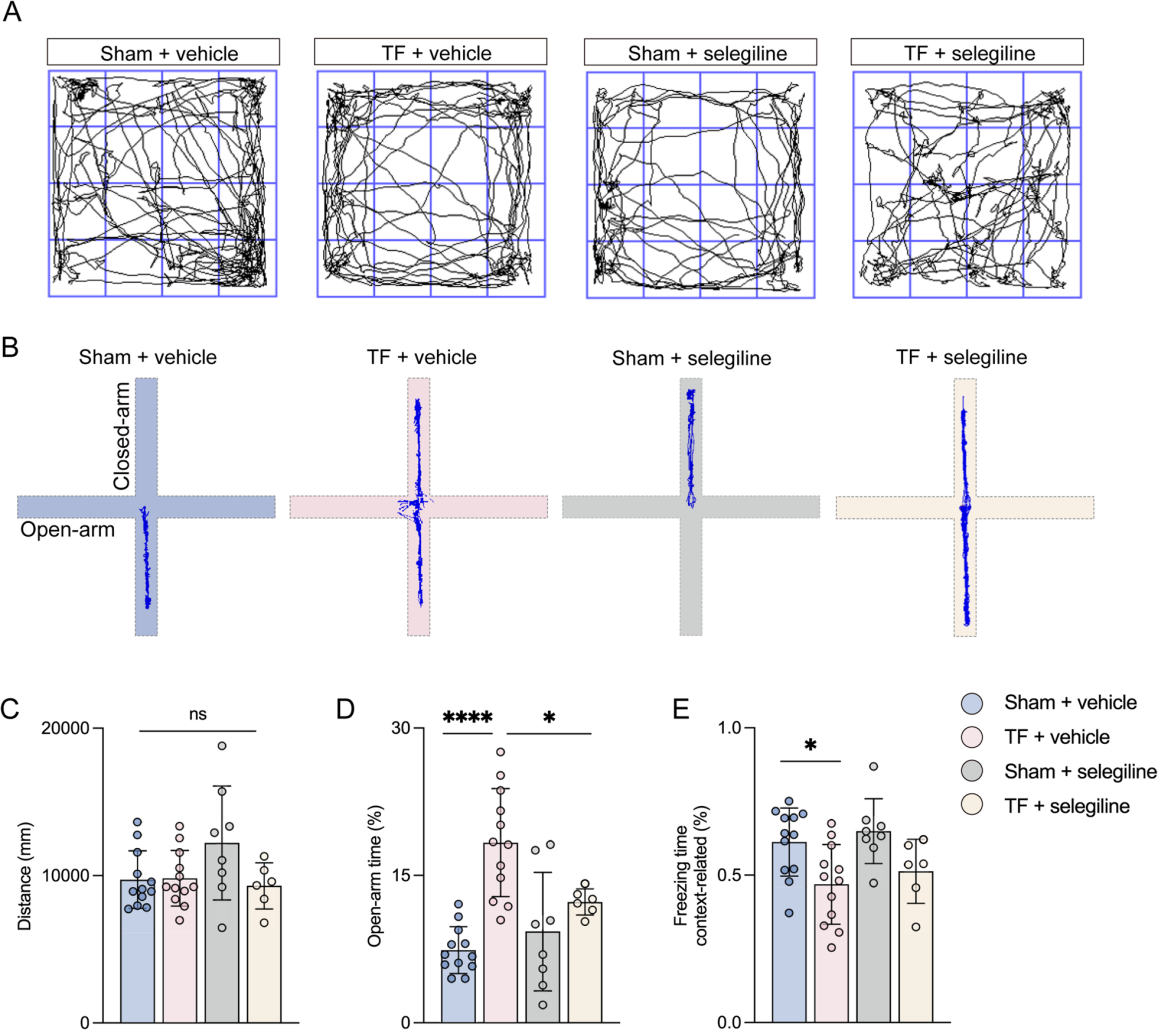
Selegiline administration ameliorates impulsive-like behaviors but not cognitive impairment post-surgery. **A** Computer printouts showing the shifting trajectories of each group in the OFT at 8 days after surgical exposure. **B** Computer printouts showing the shifting trajectories of each group in the EPM at 8 days after surgical exposure. **C** The total distance for each group at 8 days after surgical exposure. **D** The time spent in the open arm for each group at 8 days after surgical exposure. **E** The freezing time caused by the indicated stimuli during the FC test results. Data are presented as the mean ± SD (*n* = 12 mice/group). Data were analyzed by one-way ANOVA with Tukey’s multiple comparison test or Kruskal–Wallis and Dunn’s multiple comparison test. ****P < 0.0001; *P < 0.05

Our results showed that selegiline administration significantly lowered the duration in the open arm in aged mice exposed to TF plus selegiline compared with TF plus vehicle exposure (Fig. 4B, D). However, there was no significant difference in context-related freezing time in the FC between aged mice administered with selegiline and vehicle post-surgery (Fig. 4E). Besides, overall distance and average speed in the OFT did not differ significantly between the four groups (Fig. 4A, C). These data signaled that selegiline administration ameliorated impulsive-like behaviors but not cognitive impairment post-surgery.

**Fig. 5:**
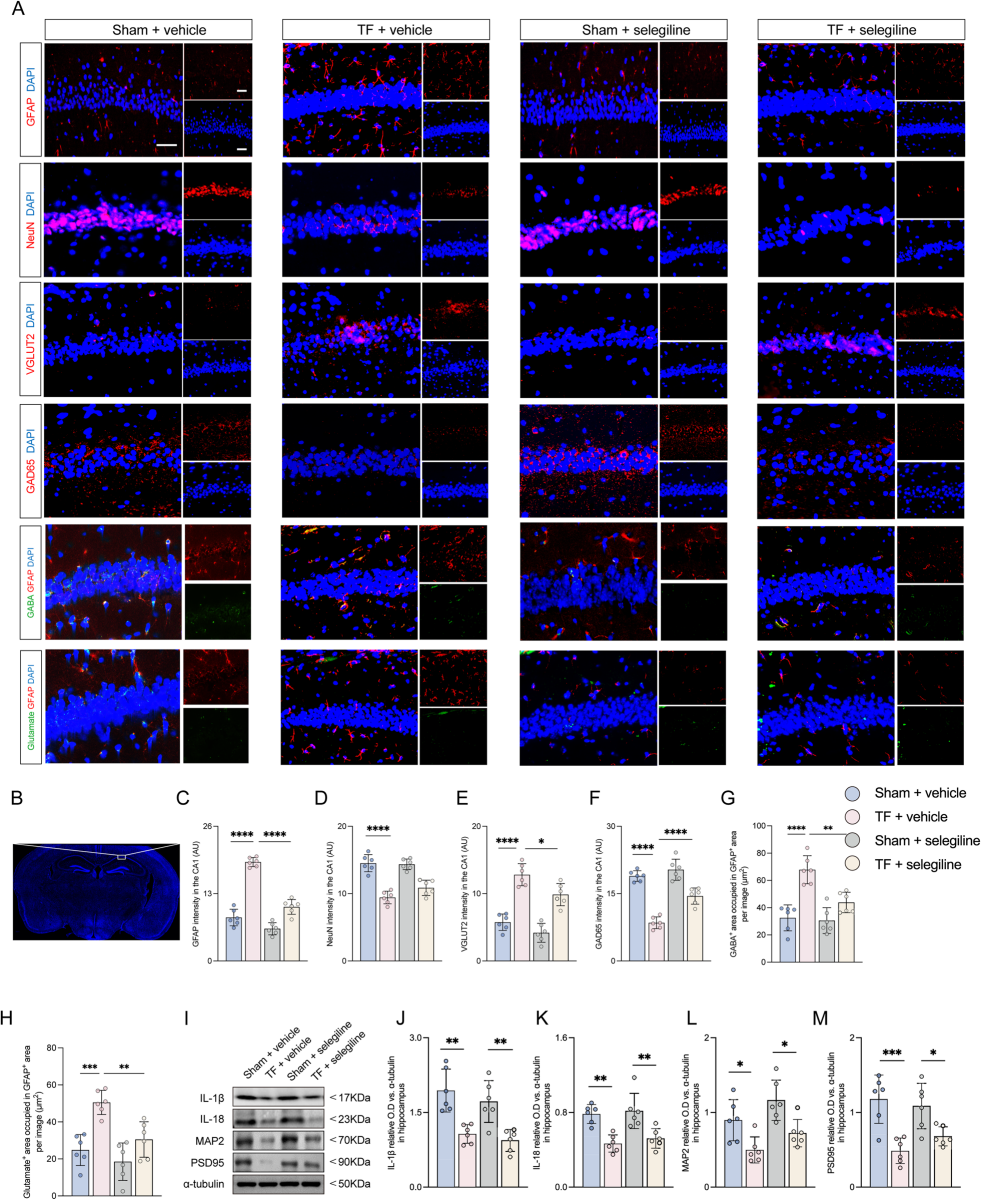
The imbalance between excitatory and inhibitory neurons can be restored by selegiline administration. **A** Representative photomicrographs of GFAP-, NeuN-, VGLUT2-, GAD65-, GABA- and glutamate-positive cells in the CA1 of hippocampus at 10 days after surgical exposure. Scale bar = 50 μm. **B** Image of CA1 in hippocampus. **C**–**F** The intensity of GFAP, NeuN, VGLUT2, and GAD65 was quantified in each group. **G**, **H** Co-stained area of GABA- and GFAP-positive cells, and glutamate- and GFAP-positive cells in the CA1 of hippocampus. **I** Representative western blot of IL-1β, IL-18, MAP2, and PSD95. **J**–**M** The optical density values of IL-1β, IL-18, MAP2, and PSD95. Data are presented as the mean ± SD (*n* = 6 mice/group). Data were analyzed by one-way ANOVA with Tukey’s multiple comparison test or Kruskal–Wallis and Dunn’s multiple comparison test. ****P < 0.0001; ***P < 0.001; **P < 0.01; *P < 0.05

The histopathological results uncovered that the intensity of GFAP and VGlut2, GABA-, and glutamate-occupied regions in GFAP-positive cells were considerably reduced, but GAD65 intensity was increased in aged mice exposed to TF plus selegiline compared with TF plus vehicle exposure at 10 days after surgery (Fig. 5A–C and E–H). However, there was no significant difference in NeuN intensity (Fig. 5A, D). The western blot results demonstrated that there was no significant difference in the expression of IL-1β, IL-18, MAP2, and PSD95 in the TF plus selegiline group as compared to the TF plus vehicle group (Fig. 5I–M). These data revealed that the imbalance between excitatory and inhibitory neurons could be restored by selegiline administration, subsequently alleviating impulsive-like behaviors post-surgery.

**Fig. 6:**
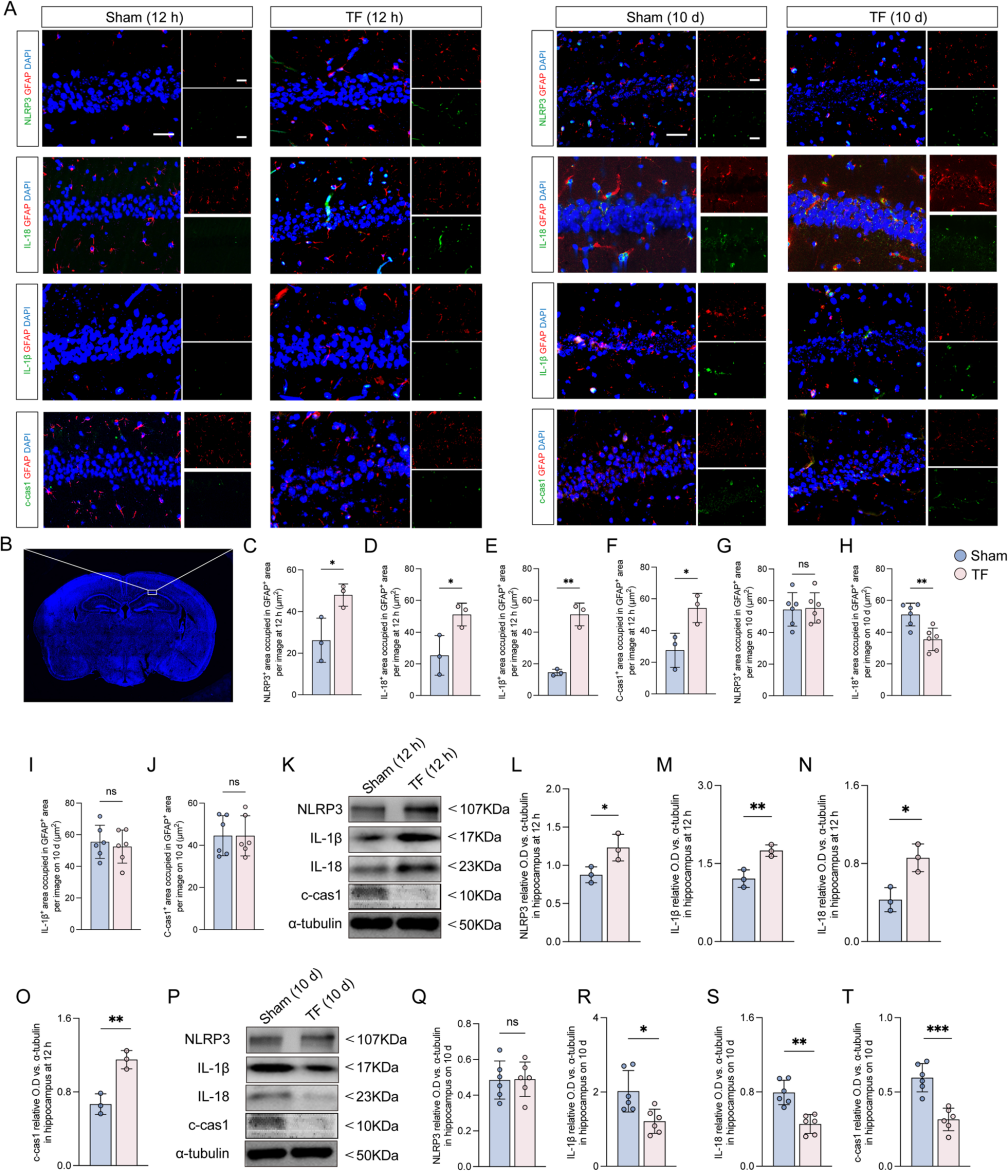
Cognitive impairment may be related to pyroptosis-associated inflammatory responses in astrocytes. **A** Representative photomicrographs of NLRP3-, IL-18-, IL-1β- and cleaved caspase-1-positive cells in the CA1 of the hippocampus at 12 h and 10 days after surgical exposure. Scale bar = 50 μm. **B** Image of CA1 in hippocampus. **C**–**J** Co-stained area of NLRP3-, IL-18-, IL-1β-, cleaved caspase-1- and GFAP-positive cells in the CA1 of the hippocampus. **K** Representative western blot of NLRP3, IL-1β, IL-18 and cleaved caspase-1 at 12 h after surgical exposure. **L**–**O** The optical density values of NLRP3, IL-1β, IL-18, and cleaved caspase-1 at 12 h after surgical exposure. **P** Representative western blot of NLRP3, IL-1β, IL-18 and cleaved caspase-1 at 10 days after surgical exposure. **Q**–**T** The optical density values of NLRP3, IL-1β, IL-18 and cleaved caspase-1 at 10 days after surgical exposure. Data are presented as the mean ± SD (*n* = 6 mice/group or *n* = 3 mice/group). Data were analyzed by one-way ANOVA with Tukey’s multiple comparison test or Kruskal–Wallis and Dunn’s multiple comparison test. ***P < 0.001; **P < 0.01; *P < 0.05

Furthermore, we investigated pyroptosis-associated inflammatory factors, including IL-1β, cleaved caspase-1, and IL-18 at 12 h and 10 days after surgery. The results exposed that TF exposure significantly increased NLRP3 expression levels in the astrocyte and upregulated IL-1β, cleaved caspase-1, and IL-18 expressions in astrocytes compared to sham at 12 h after surgery (Fig. 6A–F). Notably, the expression levels of NLRP3, IL-1β, cleaved caspase-1, and IL-18 were significantly upregulated in the hippocampus compared to the sham at 12 h (Fig. 6K–O). In contrast, there were no changes in the expression levels of NLRP3, IL-1β, cleaved caspase-1 in astrocytes between mice exposed to TF and sham at 10 days after surgery (Fig. 6A and G–J). In addition, there was a decrease in expression levels of IL-18 in the hippocampus compared to the sham (Fig. 6A, H). Nevertheless, the expression levels of IL-1β, cleaved caspase-1, and IL-18 in the hippocampus were significantly decreased compared with the sham group 10 days after surgery, and the expression of NLRP3 was not significantly different compared with the sham group (Fig. 6P–T). Earlier studies described that normal levels of IL-18 and IL-1β contribute to synaptic formation [24, 25]. These data revealed that cognitive impairment may be related to pyroptosis-associated inflammatory responses in astrocytes.

### Astrocyte-specific NLRP3 knockout ameliorates impulsive-like behaviors and cognitive impairment post-surgery in aged mice

**Fig. 7:**
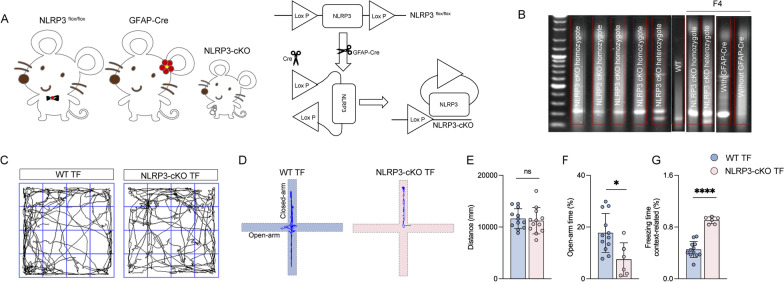
Astrocyte-specific NLRP3 knockout ameliorates impulsive-like behaviors and cognitive impairment post-surgery. **A** Breeding scheme for GFAP-Cre mice crossed with NLRP3-cKO mice and gene strategy for preparing NLRP3-conditional knockout (cKO) mice. **B** Representative genotyping analysis for NLRP3-cKO homozygote, NLRP3-cKO heterozygote, and WT (wild type) mice and NLRP3-cKO homozygote, NLRP3-cKO heterozygote, GFAP-Cre, non-GFAP-Cre F4 mice. **C** Computer printouts showing the shifting trajectories of each group in the OFT at 8 days after surgical exposure. **D** Computer printouts showing the shifting trajectories of each group in the EPM at 8 days after surgical exposure. **E** The total distance for each group at 8 days after surgical exposure. **F** The time spent in the open arm for each group at 8 days after surgical exposure. **G** The freezing time caused by the indicated stimuli during the FC test results. Data are presented as the mean ± SD (*n* = 12 mice/group). Data were analyzed by one-way ANOVA with Tukey’s multiple comparison test or Kruskal–Wallis and Dunn’s multiple comparison test. ****P < 0.0001; *P < 0.05

Our results indicated elevated levels of pyroptosis-related inflammatory factors at an early stage in the TF model. Therefore, astrocyte conditional knockout NLRP3 mice were generated for subsequent experiments using the strategy of breeding NLRP3^flox/flox^ mice with GFAP-Cre mice (Fig. 7A, B). It was shown that astrocyte-specific NLRP3 knockout mice exhibited significant decrease of duration in the open arm and an increase in context-related freezing time in the FC compared to wild-type at 8 days post-surgery (Fig. 7D, F, G). Overall distance and average speed in the OFT did not differ significantly between the two groups (Fig. 7C, E). Taken together, these data suggest that astrocyte-specific NLRP3 knockout ameliorates impulsive-like behaviors and cognitive impairment post-surgery.

**Fig. 8:**
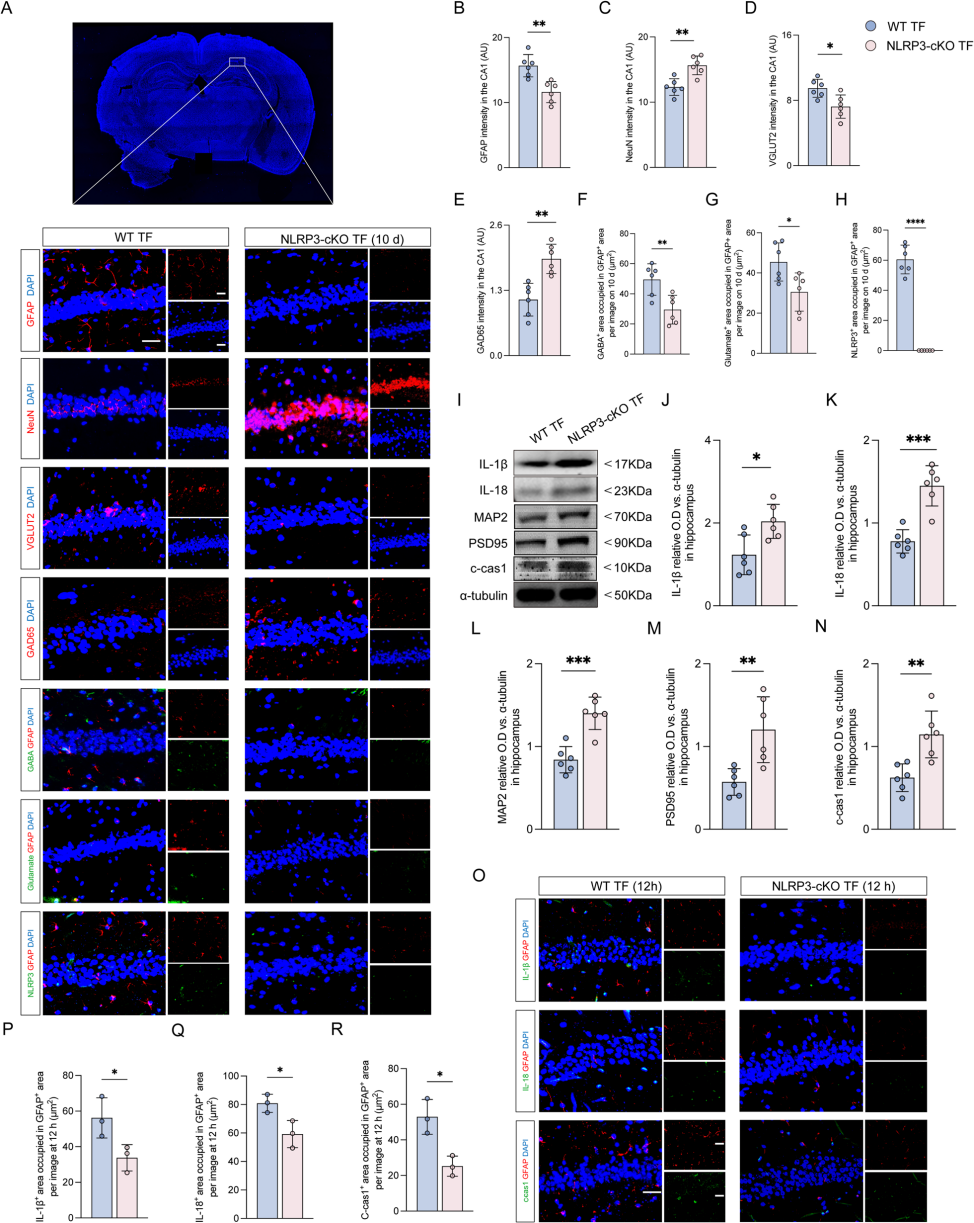
Astrocyte-specific NLRP3 knockout mitigated reactive astrocytes and excessive GABA, and improved neuronal degeneration. **A** Image of CA1 in hippocampus. Representative photomicrographs of GFAP-, NeuN-, VGLUT2-, GAD65-, GABA-, glutamate- and NLRP3-positive cells in the CA1 of hippocampus at 10 days after surgery. Scale bar = 50 μm. **B**–**E** The intensity of GFAP, NeuN, VGLUT2 and GAD65 was quantified in each group. **F**–**H** Co-stained area of GABA-, glutamate-, and NLRP3- and GFAP-positive cells in the CA1 of the hippocampus. **I** Representative western blot of IL-1β, IL-18, MAP2, PSD95 and cleaved caspase-1 at 10 days after surgery. **J**–**N** The optical density values of IL-1β, IL-18, MAP2, PSD95, and cleaved caspase-1. **O** Representative photomicrographs of IL-1β-, IL-18- and cleaved caspase-1-positive cells in the CA1 of the hippocampus at 12 h after surgical exposure. Scale bar = 50 μm. **P**–**R** Co-stained area of IL-1β-, IL-18-, cleaved caspase-1- and GFAP-positive cells in the CA1 of the hippocampus. Data are presented as the mean ± SD (*n* = 6 mice/group or *n* = 3 mice/group). Data were analyzed by one-way ANOVA with Tukey’s multiple comparison test or Kruskal–Wallis and Dunn’s multiple comparison test. ****P < 0.0001; ***P < 0.001; **P < 0.01; *P < 0.05

The histopathological results showed that the intensity of GFAP and VGlut2, GABA-, and glutamate-occupied regions in GFAP-positive cells were significantly reduced, but GAD65 and NeuN intensity (Fig. 8A–G). Moreover, the expression of NLRP3 in astrocytes could not be detected in the hippocampal region of the astrocyte-specific NLRP3 knockout mice (Fig. 8A, H). The result of western blot revealed that the expressions of IL-1β, IL-18, cleaved caspase-1, MAP2, and PSD95 in the hippocampus were increased in astrocyte-specific NLRP3 knockout mice exposed to TF compared with wild-type at 10 days after surgery (Fig. 8I–N). In addition, we found significant decreases in the expression levels of pyroptosis-associated inflammatory factors, including IL-1β, IL-18, and cleaved caspase-1 in the astrocyte at 12 h after surgery (Fig. 8O–R).

## Discussion

In the current study, we provided compelling evidence of the vital role of the NLRP3–GABA signaling pathway in anesthesia and surgery-induced PND. Selegiline administration significantly ameliorated TF-induced impulsive-like behaviors but not cognitive impairment and lowered abundant GABA levels in reactive astrocytes in the hippocampus. Moreover, astrocyte-specific NLRP3 knockout mice reversed TF-induced impulsive-like and cognitive impairment behaviors, decreased GABA levels in reactive astrocytes, ameliorated NLRP3-associated inflammatory responses at the early stage, and restored neuronal degeneration in the hippocampus. We completed the EPM and FC training on the same day, and the results obtained for FC are consistent with those of Lai et al. suggesting that the effect of EPM on FC is negligible [26]. Collectively, our data revealed that the inhibition of NLRP3–GABA signaling pathways in astrocytes has huge prospects for clinical implementation to assist in alleviating PND in the clinical setting.

Several rodent models, including tibial fracture, carotid artery exposure, and exploratory open abdominal surgery, have been considered to simulate PND conditions [27–29]. Previous publications demonstrated that aged rodents, after surgical exposure, exhibit cognitive dysfunction, as indicated by decreases in context-related freezing time, which is in line with our present study [30, 31]. Interestingly, we also found tibial fracture surgery induced significant impulsive-like behaviors, which manifest as delirium-like behaviors in the clinical setting [32]. Neuronal loss in the hippocampus is reportedly involved in the course of PND, which is in line with the observations of our current study [33, 34]. Besides, reactive astrocytes, lower GABA levels, glutamate in astrocytes, decreased GAD65 activity, and increased VGlut2 intensity were detected in the hippocampus, subsequently leading to an imbalance in inhibitory and excitatory signals. These findings add to the evidence that imbalances in inhibitory and excitatory signals contributed to memory dysfunction and impulsive behaviors [35, 36]. Therefore, these observations imply that impulsive-like behaviors and cognitive impairment seen in aged mice post-surgery are probably attributable to excessive GABA activity in astrocytes.

Increased tonic GABA release by reactive astrocytes in the hippocampus exerts memory dysfunction [12]. Excess GABA in reactive astrocytes can suppress the activity of nearby interneurons, leading to the de-inhibition of glutamatergic principal neurons and resulting in previously reported epileptiform discharges [37]. According to the previous study, selegiline was effective in reducing GABA synthesis by astrocytes [12]. Our findings suggested that selegiline administration significantly relieved TF-induced impulsive-like behaviors, but appeared to have minimal improvement in cognitive function. The improvement in impulsive behavior may be related to the fact that selegiline reduced astrocyte-derived GABA. Selegiline administration was carried out from day 1 to day 7 post-surgery, and failed to affect early inflammatory responses within 24 h after surgery. However, cognitive dysfunction has been reported to be associated with inflammatory responses at the early stage after surgery [38]. Furthermore, knockdown of NLRP3 in astrocytes not only reduced GABA production in astrocytes, but also increased neuronal survival and improved neuronal function, thereby ameliorating not only TF-induced impulse-like behaviors but also cognitive impairment. We also found that the upregulation of NLRP3-associated inflammatory factors at the early stage following surgical exposure and the downregulation at the late stage participated in the process of cognitive impairment. It was demonstrated that an early inflammatory response occurs in astrocytes, leading to astrocytic activation and excessive GABA production [39]. Thus, these observations signaled that selegiline can ameliorate impulsive-like behaviors but not mitigate early inflammatory response in astrocytes, further contributing to cognitive impairment.

NLRP3 is implicated in the initiation of immune-inflammatory responses and in the formation of inflammatory vesicles, thereby promoting the maturation and secretion of pro-inflammatory cytokines, such as IL-1β and IL-18 [40]. Interestingly, we have previously reported that the deletion of NLRP3 in astrocytes mitigates neuroinflammation at the early stage after traumatic stress [17]. In the present study, we found that NLRP3 expression in astrocytes was upregulated at the early stage, subsequently leading to the upregulation of cleaved caspase-1, IL-1β, and IL-18. More importantly, the deletion of NLRP3 in astrocytes not only mitigated reactive astrocytes and excessive GABA, but also ameliorated neuronal degeneration. There is a growing body of evidence reporting that targeting NLRP3-mediated inflammation in astrocytes may provide potential therapeutic benefits for the pathogenesis of depression [41, 42]. In addition, the levels of IL-1β and IL-18 in the hippocampus were restored at the late stage after surgical exposure by the deletion of NLRP3 in astrocytes. Prior publications suggested IL-1β and IL-18-induced effects on synaptic plasticity and functionality within the hippocampal system [43, 44]. Our further analysis of the results revealed that the expression levels of IL-1β and IL-18 in the sham-operated group were higher on postoperative day 10 than at 12 h postoperatively, which may be due to the fact that the mice underwent behavioral tests including OFT, EPM, and FC on postoperative days 8 and 9, and that electrical stimulation of FC in particular may have led to elevated levels of inflammation in the mice. This further suggests that the level of inflammation and cognitive function are correlated. Surprisingly, a normal level of inflammatory factors in the brain plays a vital role in neuronal differentiation and neurite branching [45].

There are some limitations and shortcomings in this study. Firstly, we found a significant increase in GABA expression in astrocytes. In our previous study, we have found that NLRP3 in astrocytes has a significant ameliorating effect on perioperative traumatic stress [17]. However, whether NLRP3 in microglia can play a compensatory role is unknown and will be further explored in the future. Secondly, we have only semi-quantified astrocyte-derived GABA using immunofluorescence. Next, we will use in vivo GABA probes or in vivo cranial microdialysis combined with mass spectrometry for precise quantification. Thirdly, the relationship between anesthesia and impulsive-like behavior is unclear. We will continue to explore in depth the relationship between anesthesia and impulse-like behavior in the future.

Taken together, this current study suggests that anesthesia and surgical exposure in aged mice cause impulsive-like behaviors and cognitive impairment, which may be associated with increased NLRP3 activity in astrocytes. The current study helps to clarify the therapeutic process of PND and provides novel ideas for targeting signals to against PND.

## Supplementary Information

**Additional file 1: Table S1.** Table of the number of mice used during the experiment. **Figure S1.** Original image of protein immune bands. (A) Original images of western blot of protein IL-1β. (B) Original images of western blot of protein IL-18. (C) Original images of western blot of protein cleaved-caslase-1. (D) Original images of western blot of protein PSD95. (E) Original images of western blot of protein MAP2. (F) Original images of western blot of protein α-Tubulin. (G) Original images of western blot of protein NLRP3. **Figure S2.** The results of the Morris water maze experiment in two groups of mice in the pre-experiment. (A) Representative water maze trajectories of mice in Sham and TF groups. (B) The number of water Morris maze cross-platform for two groups of mice. (C) The total distance of Morris water maze in two groups of mice. (D) Morris water maze escape latency of two groups of mice. (E) The time spent in the target quadrant of two groups of mice. Data are presented as the mean ± SD (*n* = 10 mice/group). Data were analyzed by one-way ANOVA with Tukey’s multiple comparison test or Kruskal–Wallis and Dunn’s multiple comparison test. **Figure S3.** Weight gain curves of WT and NLRP3-cKO mice. **Figure S4.** Astrocytes-specific NLRP3 knockout in combination with selegiline administration did not significantly improve impulse-like behaviors and cognitive dysfunction after TF surgery. (A) Computer printouts showing the shifting trajectories of each group in the OFT at 8 days after surgical exposure. (B) The total distance for each group at 8 days after surgical exposure. (C) Computer printouts showing the shifting trajectories of each group in the EPM at 8 days after surgical exposure. (D) The time spent in the open arm for each group at 8 days after surgical exposure. (E) Freezing time during the FC test results caused by the indicated stimuli. Data are presented as the mean ± SD (*n* = 12 mice/group). Data were analyzed by one-way ANOVA with Tukey’s multiple comparison test or Kruskal–Wallis and Dunn’s multiple comparison test. ****P < 0.0001; *P < 0.05. **Figure S5.** Changes in excitatory and inhibitory neurons in the CA3 region of the hippocampus in each group. (A) Image of CA1 in hippocampus. (B) Representative photomicrographs of VGLUT2 and GAD65 in the CA3 of the hippocampus. Scale bar = 25 μm. (C, D) The intensity of VGLUT2 and GAD65 was quantified in each group. (E) Representative photomicrographs of VGLUT2 and GAD65 in the CA3 of the hippocampus. Scale bar = 25 μm. (F, G) The intensity of VGLUT2 and GAD65 was quantified in each group. (H) Representative photomicrographs of VGLUT2 and GAD65 in the CA3 of the hippocampus. Scale bar = 25 μm. (I, J) The intensity of VGLUT2 and GAD65 was quantified in each group. Data are presented as the mean ± SD (*n* = 6 mice/group). Data were analyzed by one-way ANOVA with Tukey’s multiple comparison test or Kruskal–Wallis and Dunn’s multiple comparison test. ****P < 0.0001; ***P < 0.001; **P < 0.01; *P < 0.05.

## Data Availability

The datasets used and/or analyzed during the present study are available from the corresponding author upon reasonable request.
