# Supplementary material for: NLRP3–GABA signaling pathway contributes to the pathogenesis of impulsive-like behaviors and cognitive deficits in aged mice
Source: J Neuroinflammation. 2023 Jul 11;20:162. doi: 10.1186/s12974-023-02845-3 (PMC10337164; doi:10.1186/s12974-023-02845-3)
Supplement: Supplementary file 1 — Additional file 1: Table S1. Table of the number of mice used during the experiment. Figure S1. Original image of protein immune bands. (A) Original images of western blot of protein IL-1β. (B) Original images of western blot of protein IL-18. (C) Original images of western blot of protein cleaved-caslase-1. (D) Original images of western blot of protein PSD95. (E) Original images of western blot of protein MAP2. (F) Original images of western blot of protein α-Tubulin. (G) Original images of western blot of protein NLRP3. Figure S2. The results of the Morris water maze experiment in two groups of mice in the pre-experiment. (A) Representative water maze trajectories of mice in Sham and TF groups. (B) The number of water Morris maze cross-platform for two groups of mice. (C) The total distance of Morris water maze in two groups of mice. (D) Morris water maze escape latency of two groups of mice. (E) The time spent in the target quadrant of two groups of mice. Data are presented as the mean ± SD (n = 10 mice/group). Data were analyzed by one-way ANOVA with Tukey’s multiple comparison test or Kruskal–Wallis and Dunn’s multiple comparison test. Figure S3. Weight gain curves of WT and NLRP3-cKO mice. Figure S4. Astrocytes-specific NLRP3 knockout in combination with selegiline administration did not significantly improve impulse-like behaviors and cognitive dysfunction after TF surgery. (A) Computer printouts showing the shifting trajectories of each group in the OFT at 8 days after surgical exposure. (B) The total distance for each group at 8 days after surgical exposure. (C) Computer printouts showing the shifting trajectories of each group in the EPM at 8 days after surgical exposure. (D) The time spent in the open arm for each group at 8 days after surgical exposure. (E) Freezing time during the FC test results caused by the indicated stimuli. Data are presented as the mean ± SD (n = 12 mice/group). Data were analyzed by one-way ANOVA with Tukey’s multiple comparison [file 12974_2023_2845_MOESM1_ESM.docx]

**Additional file 1**

**Table S1**

| **Group** | **Total** | **Death** | **For IF** | | **For WB** | | **For behavioral** |
| --- | --- | --- | --- | --- | --- | --- | --- |
|  |  |  | **12 h** | **10 d** | **12 h** | **10 d** | **8-9 d** |
| **Sham**  **(n =30)** | 18 | 0 | 3 | 6 | 3 | 6 | 12 |
| **TF**  **(n =32)** | 20 | 2 | 3 | 6 | 3 | 6 | 12 |
| **Sham + vehicle**  **(n =21)** | 12 | 0 | 0 | 6 | 0 | 6 | 12 |
| **TF + vehicle**  **(n =21)** | 12 | 0 | 0 | 6 | 0 | 6 | 12 |
| **Sham + selegiline**  **(n =17)** | 12 | 0 | 0 | 6 | 0 | 6 | 8 |
| **TF + selegiline**  **(n =16)** | 13 | 1 | 0 | 6 | 0 | 6 | 6 |
| **WT TF**  **(n =21)** | 15 | 0 | 3 | 6 | 0 | 6 | 12 |
| **NLRP3-KO TF**  **(n =15)** | 15 | 0 | 3 | 6 | 0 | 6 | 12 |

**Table of the number of mice used during the experiment. IF:** immunofluorescence, **WB:** western

blot, **Vehicle:** sterilized saline, **WT:** wild type,**NLRP3 KO:** NLRP3 knockout in astrocytes


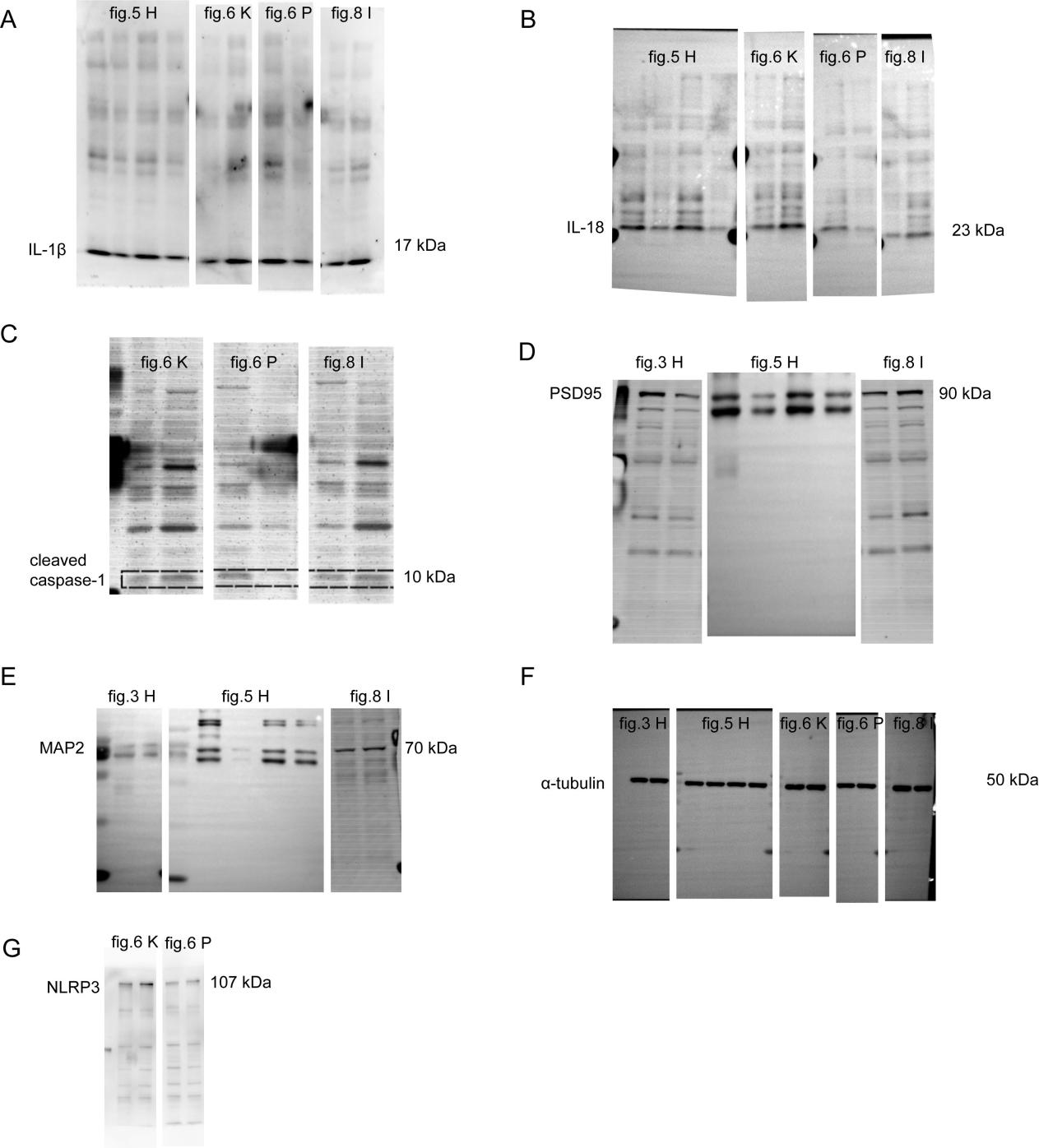


**Figure S1**

**Original image of protein immune bands.** (A) Original images of western blot of protein IL-1β. (B) Original images of western blot of protein IL-18. (C) Original images of western blot of protein cleaved-caslase-1. (D) Original images of western blot of protein PSD95. (E) Original images of western blot of protein MAP2. (F) Original images of western blot of protein α-Tubulin. (G) Original images of western blot of protein NLRP3.


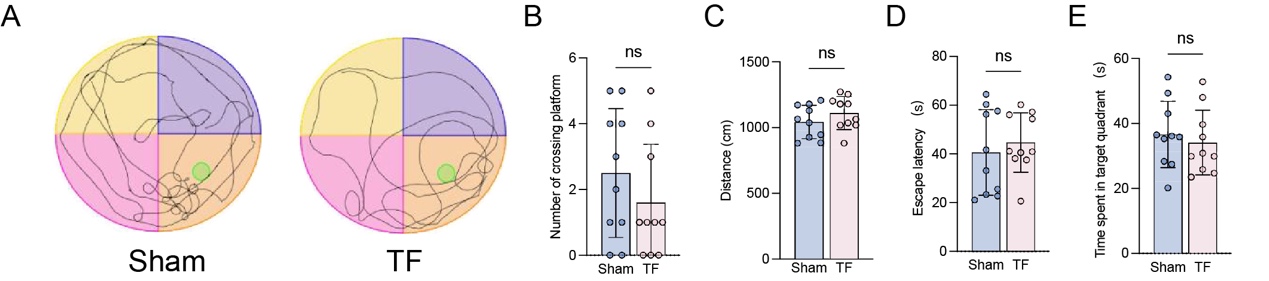


**Figure S2**

**The results of the Morris water maze experiment in two groups of mice in the pre-experiment.** (A) Representative water maze trajectories of mice in Sham and TF groups. (B) The number of water Morris maze cross-platform for two groups of mice. (C) The total distance of Morris water maze in two groups of mice. (D) Morris water maze escape latency of two groups of mice. (E) The time spent in the target quadrant of two groups of mice. Data are presented as the mean ± SD (n = 10 mice/group). Data were analyzed by one-way ANOVA with Tukey's multiple comparison test or Kruskal–Wallis and Dunn's multiple comparison test.


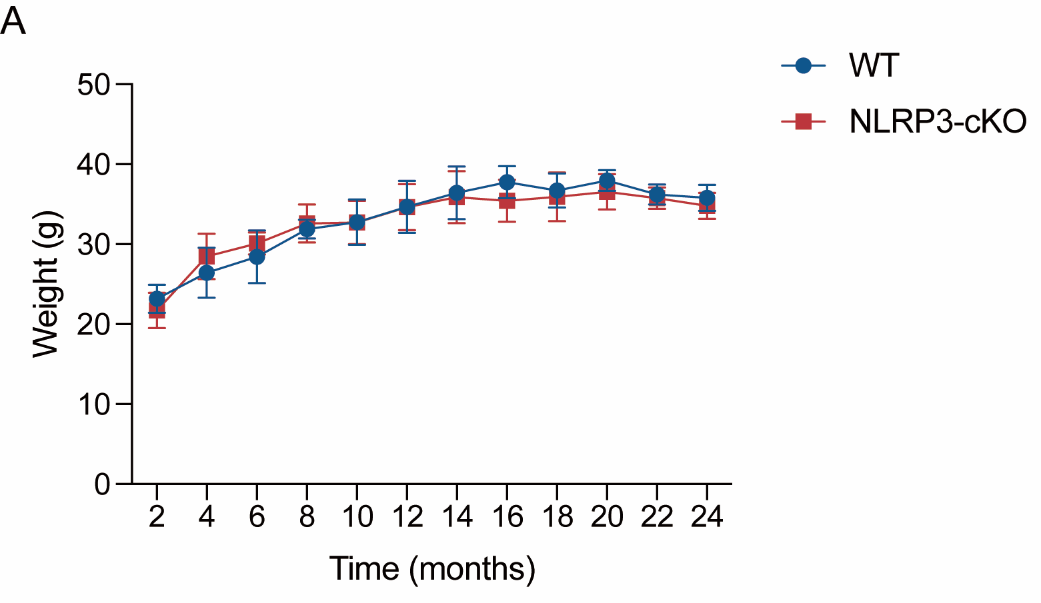


**Figure S3**

**Weight gain curves of WT and NLRP3-cKO mice.**

**
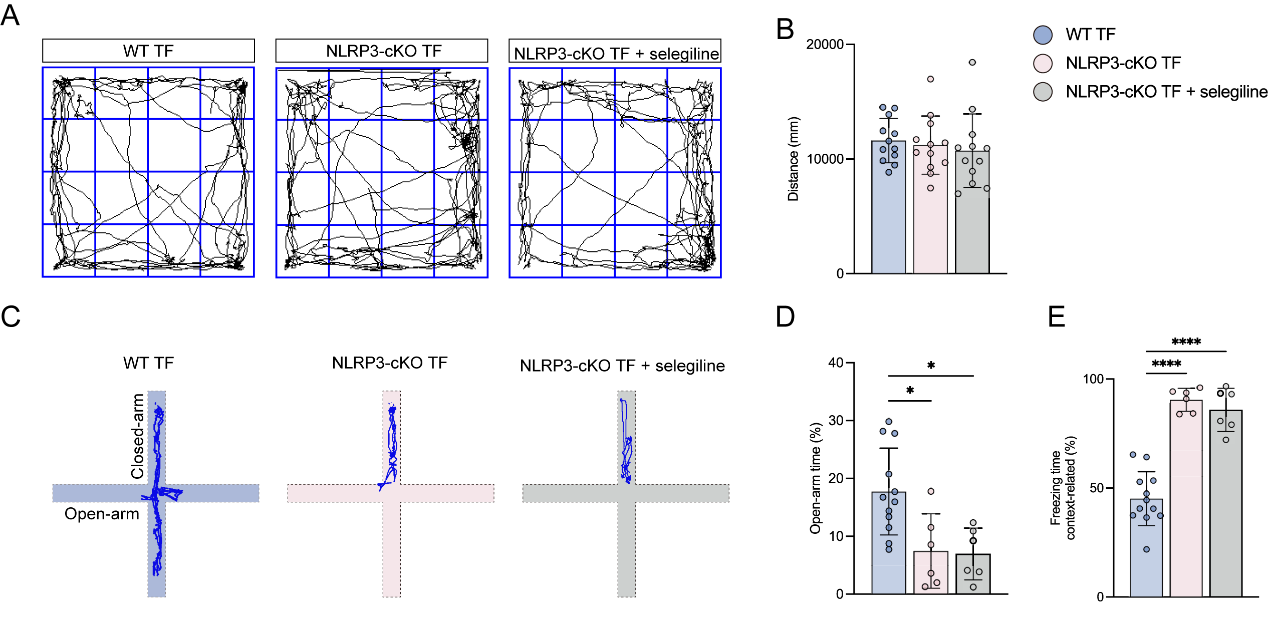
****Figure S4**

**Astrocytes-specific NLRP3 knockout in combination with selegiline administration did not significantly improve impulse-like behaviors and cognitive dysfunction after TF surgery.** (A) Computer printouts showing the shifting trajectories of each group in the OFT at 8 days after surgical exposure. (B) The total distance for each group at 8 days after surgical exposure. (C) Computer printouts showing the shifting trajectories of each group in the EPM at 8 days after surgical exposure. (D) The time spent in the open arm for each group at 8 days after surgical exposure. (E) Freezing time during the FC test results caused by the indicated stimuli. Data are presented as the mean ± SD (n = 12 mice/group). Data were analyzed by one-way ANOVA with Tukey's multiple comparison test or Kruskal–Wallis and Dunn's multiple comparison test. *****P* < 0.0001; **P* < 0.05.


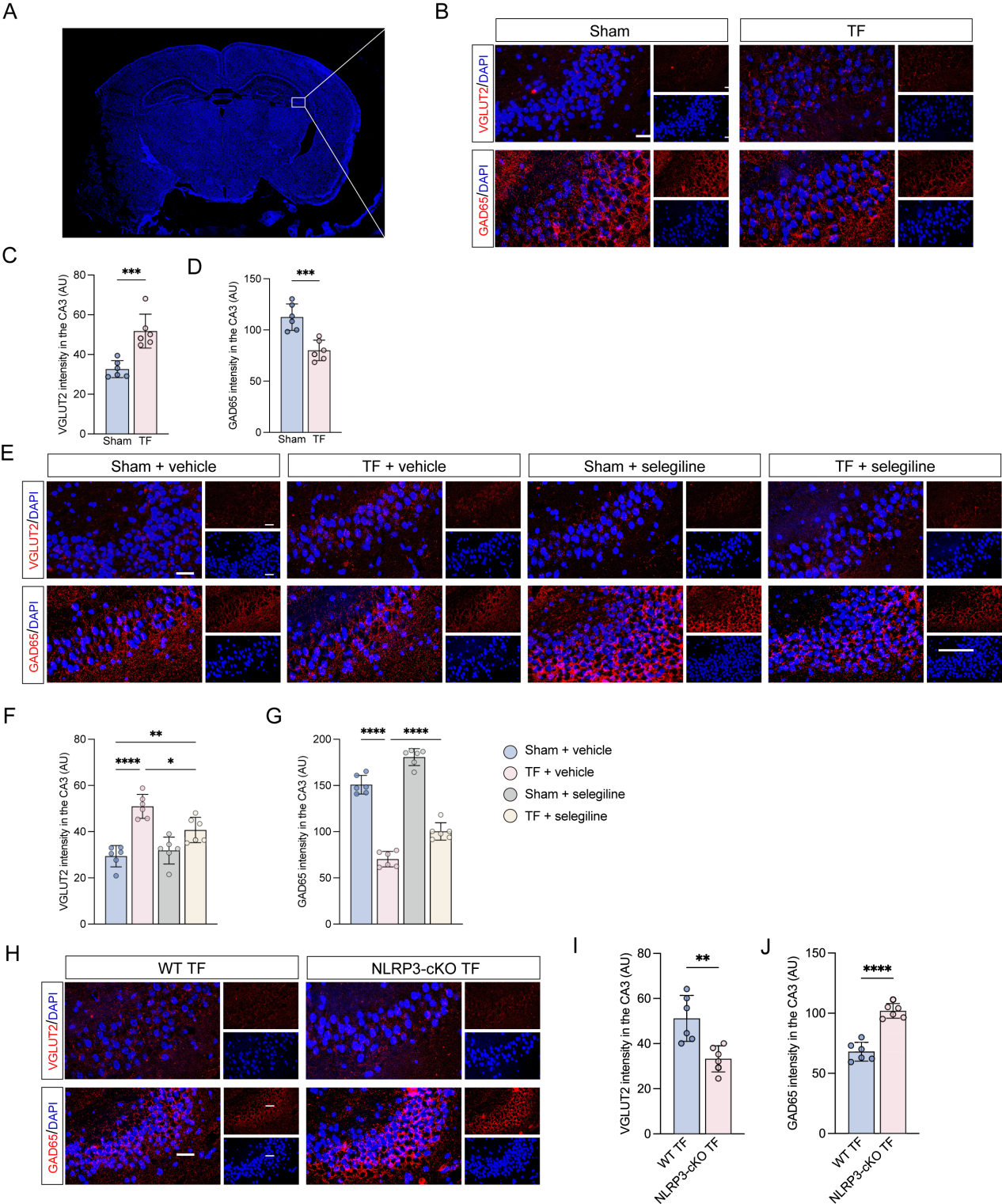


**Figure S5**

**Changes in excitatory and inhibitory neurons in the CA3 region of the hippocampus in each group.** (A) Image of CA1 in hippocampus. (B) Representative photomicrographs of VGLUT2 and GAD65 in the CA3 of the hippocampus. Scale bar = 25 μm. (C-D) The intensity of VGLUT2 and GAD65 was quantified in each group. (E) Representative photomicrographs of VGLUT2 and GAD65 in the CA3 of the hippocampus. Scale bar = 25 μm. (F-G) The intensity of VGLUT2 and GAD65 was quantified in each group. (H) Representative photomicrographs of VGLUT2 and GAD65 in the CA3 of the hippocampus. Scale bar = 25 μm. (I-J) The intensity of VGLUT2 and GAD65 was quantified in each group. Data are presented as the mean ± SD (n = 6 mice/group). Data were analyzed by one-way ANOVA with Tukey's multiple comparison test or Kruskal–Wallis and Dunn's multiple comparison test. *****P* < 0.0001; ****P* < 0.001; ***P* < 0.01; **P* < 0.05.
